# Impact of critical illness on cholesterol and fatty acids: insights into pathophysiology and therapeutic targets

**DOI:** 10.1186/s40635-023-00570-y

**Published:** 2023-11-28

**Authors:** Caroline Lauwers, Lauren De Bruyn, Lies Langouche

**Affiliations:** Clinical Division and Laboratory of Intensive Care Medicine, Department of Cellular and Molecular Medicine, KU Leuven, Herestraat 49, O&N1 Box 503, 3000 Leuven, Belgium

## Abstract

Critical illness is characterized by a hypercatabolic response encompassing endocrine and metabolic alterations. Not only the uptake, synthesis and metabolism of glucose and amino acids is majorly affected, but also the homeostasis of lipids and cholesterol is altered during acute and prolonged critical illness. Patients who suffer from critically ill conditions such as sepsis, major trauma, surgery or burn wounds display an immediate and sustained reduction in low plasma LDL-, HDL- and total cholesterol concentrations, together with a, less pronounced, increase in plasma free fatty acids. The severity of these alterations is associated with severity of illness, but the underlying pathophysiological mechanisms are multifactorial and only partly clarified. This narrative review aims to provide an overview of the current knowledge of how lipid and cholesterol uptake, synthesis and metabolism is affected during critical illness. Reduced nutritional uptake, increased scavenging of lipoproteins as well as an increased conversion to cortisol or other cholesterol-derived metabolites might all play a role in the decrease in plasma cholesterol. The acute stress response to critical illness creates a lipolytic cocktail, which might explain the increase in plasma free fatty acids, although reduced uptake and oxidation, but also increased lipogenesis, especially in prolonged critical illness, will also affect the circulating levels. Whether a disturbed lipid homeostasis warrants intervention or should primarily be interpreted as a signal of severity of illness requires further research.

## Introduction

Critical illness is a complex disease state with severe endocrine and metabolic alterations [1]. Although many of these endocrine and metabolic changes may be part of a protective acute survival response, when sustained for a prolonged period of time, these affected pathways may have detrimental consequences [1, 2]. Indeed, the hypercatabolic response in the acute phase of critical illness, with high cortisol, glucagon and catecholamine levels, with insulin and GH resistance, and with low T3 levels induces a catabolic and energy-sparing fight-or-flight state which is presumed to be an adaptive response providing the essential fuel for energy production in vital organs [1, 3]. However, when hypercatabolism persists, it results in muscle wasting and weakness, associated with impaired weaning from mechanical ventilation, delayed rehabilitation and late death [2]. In addition to ongoing hypoperfusion, hypoxia and excessive inflammation, metabolic insults such as hyperglycaemia can also cause cell damage requiring adequate clearance through autophagy to allow recovery [4, 5]. Similarly, the observed dyslipidemia of critical illness encompasses acute alterations that can be interpreted as part of the acute and adaptive survival response, but also associate with worse outcome and delayed recovery in the intensive care unit (ICU) [6–9].

Sepsis and other critical illnesses are characterized by an immediate and sustained reduction in low plasma LDL-, HDL- and total cholesterol concentrations [10–15], together with a, less pronounced, increase in plasma free fatty acids (FFA) [7–9, 16, 17]. The severity of these alterations is associated with severity of illness, but the underlying pathophysiological mechanisms that might be involved are multifactorial and only partly clarified. This narrative review aims to provide an overview of the current understanding of the impact of the observed changes in lipid and cholesterol metabolism during critical illness and the potential pathophysiological underlying mechanisms. The current and emerging therapeutic strategies aimed at restoring lipid and cholesterol disturbances in critically ill patients are also discussed. These encompass pharmacological interventions, nutritional support, and metabolic targets for novel therapeutic interventions.

## Cholesterol homeostasis during critical illness

### Circulating cholesterol and lipoproteins during critical illness

Cholesterol is vital for normal systemic and cellular functioning. It is an essential constituent of cell membranes, fulfills a role in signaling and transport, and can serve as a precursor for the synthesis of various bioactive molecules, including steroid hormones and bile acids. Emerging evidence indicates that critical illness induces significant perturbations in cholesterol homeostasis. Conditions such as major surgery and trauma, sepsis, burn wounds and liver dysfunction are characterized by low total-, LDL- and HDL-cholesterol plasma concentrations [10–13, 18]. A rapid fall in total and lipoprotein cholesterol has been observed from the onset of critical illness onwards [14]. This hypocholesterolemia is most pronounced in patients with sepsis as compared with surgery or trauma ICU patients [10, 11, 15]. For both trauma and septic patients, lipoprotein concentrations further decrease during the first days of critical illness, followed by a steady but slow recovery [15, 19]. Low serum cholesterol levels were associated with higher Acute Physiology and Chronic Health Evaluation (APACHE) III score, increased Multiple Organ Dysfunction Score (MODS), longer length of stay and increased mortality [14]. Lower serum cholesterol levels have been recently documented in critically ill patients suffering from ICU-acquired weakness (ICUAW) as compared with non-weak patients [20]. Of note, altered cholesterol concentrations within lipid rafts can affect downstream signaling pathways such as the adrenergic receptor signaling pathway and might also contribute to myocardial dysfunction in septic shock patients [21, 22].

The decrease in circulating cholesterol is thought to be part of the acute phase response, as cholesterol and its lipoprotein carriers can have immunomodulatory properties [23, 24]. VLDL, LDL and HDL lipoproteins have the ability to bind and neutralize endotoxins such as bacterial lipopolysaccharide (LPS), as well as other bacterial and viral pathogenic products [23–26]. Binding of LPS to lipoproteins interferes with the interaction of LPS on Toll-like receptors (TLRs) present on macrophages, impairs TLR signaling and modulates infection and inflammation [27]. This scavenging mechanism plays an important role in neutralizing toxins as part of the innate immune system preventing activation of TLR by pathogen-associated molecular patterns and thereby establishing a first line of defense against invading micro-organism, but how much this scavenging would affect circulating cholesterol levels has not been clarified. In addition, one might expect an increased need of cholesterol for new cell synthesis as part of the immune response, tissue repair and wound healing [28–30]. At least in cancer, such increased need has been linked to hypocholesterolemia [28]. Additionally, cholesterol might also be required for the sustained conversion to cortisol in the adrenal cortex as part of the stress response to critical illness [31]. The sicker the patient, the higher plasma cortisol concentrations are and the lower plasma cholesterol concentrations.

**Fig. 1:**
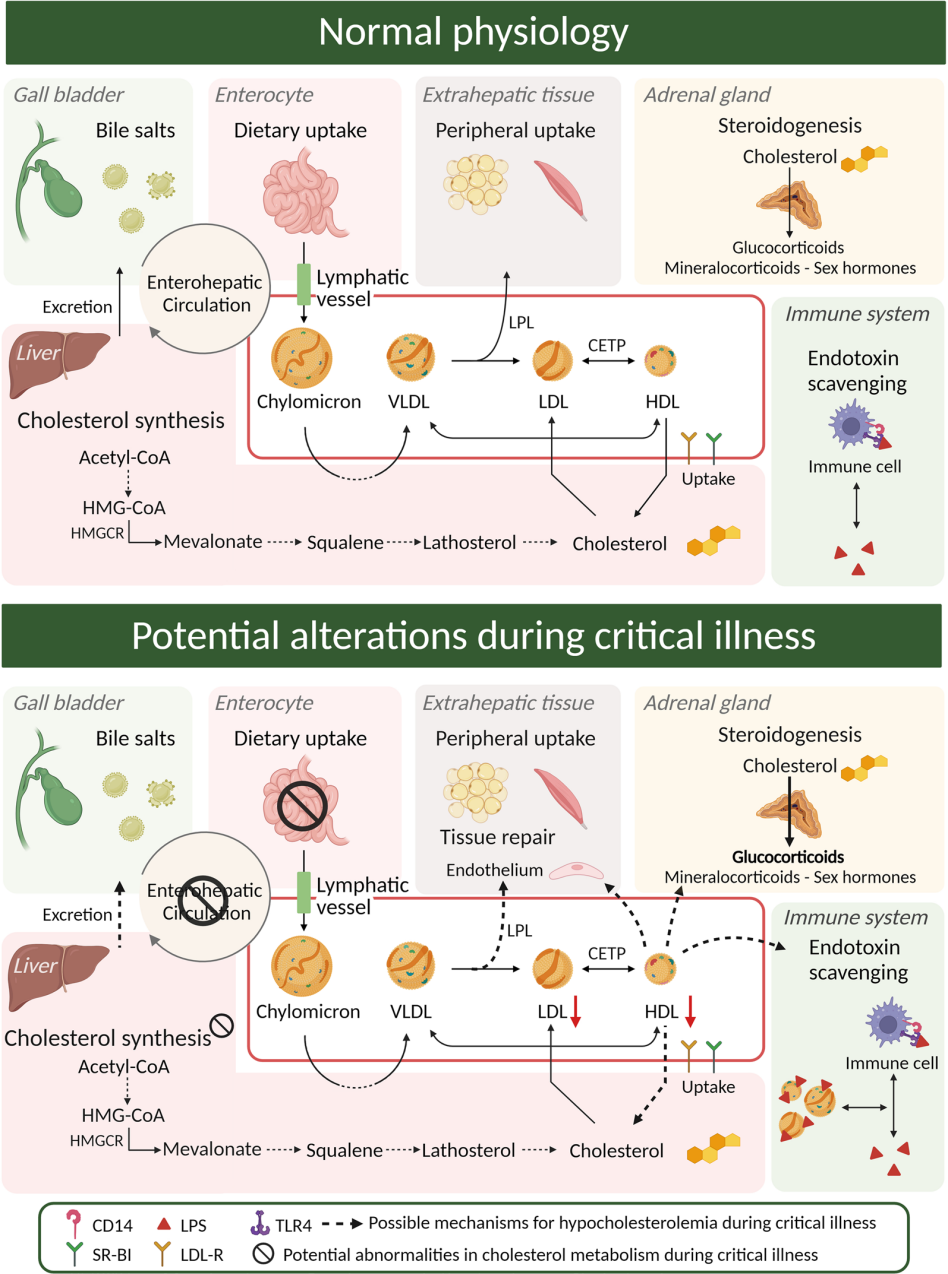
Schematic overview of normal cholesterol physiology and possible mechanisms involved cholesterol disturbances during critical illness. In normal physiology, dietary cholesterol is taken up from the intestine and stored as cytosolic lipid droplets or packed into lipoproteins to enable transport in the circulation. Cholesterol can be converted and excreted as bile acids as part of the enterohepatic circulation or converted to steroid hormones. The liver is the main organ for de novo synthesis of cholesterol from Acetyl CoA through the mevalonate pathway, for which HMGCR is the key-regulator. During critical illness, reduced dietary uptake and reduced bile acid excretion are involved in a disturbed enterohepatic circulation. Cholesterol synthesis appears reduced. Increased shuttling to tissue repair, LPS scavenging and conversion to steroid hormones might all play a role. LPS: lipopolysaccharide; TLR 4: toll-like receptor 4; CD14: cluster of differentiation 14; HMGCR: HMG-CoA reductase. SR-BI: scavenger receptor class B type I; LDL-R: LDL-receptor. Created with BioRender.com

Cholesterol homeostasis is in normal physiology tightly regulated where the amount of cholesterol taken up through the diet determines the endogenous cholesterol production (Fig. 1). In the next section, we will discuss the different components involved in cholesterol homeostasis and how they are affected during critical illness and possibly explain the hypocholesterolemia of critical illness.

### Pathophysiology

#### Uptake and transport of cholesterol during critical illness

In normal conditions, dietary cholesterol is taken up from the intestine and stored as cytosolic lipid droplets or packed into lipoproteins to enable transport in the circulation (Fig. 1). In critically ill patients however, cholesterol intake is often reduced, as the lipid fraction of enteral and parenteral formulas often lacks cholesterol, as only fish oil, but not soy or olive oil is a source of cholesterol [32, 33]. Furthermore, intestinal absorption of lipid is impaired during critical illness [34]. In addition, ATP-binding cassette transporters, transforming lipid-poor apolipoprotein A1 into mature HDL, and lecithin-cholesterol acyltransferase (LCAT), converting free cholesterol to cholesterol esters, are also affected during sepsis [35, 36]. Cholesteryl ester transfer protein (CETP), responsible for the transfer of cholesterol between HDL and LDL (Fig. 1), has been shown in animal models to be reduced by sepsis or inflammation [37]. Increased CETP activity would deplete HDL particles of their cholesterol content, induce HDL catabolism, and reduce HDL plasma concentration, which suggests that the observed decrease with sepsis is a compensatory mechanism. No human data on CETP activity is available, but a CETP gain-of-function genetic variant was associated with increased sepsis mortality [38].

#### De novo cholesterol production during critical illness

Cholesterol synthesis can occur in every nucleated cell, but the majority is synthesized in the liver in a multi-enzyme reaction which is high energy and oxygen demanding (Fig. 1). Cholesterol is synthesized in the mevalonate pathway starting from acetyl co-enzyme A (acetyl CoA), and with HMG-CoA reductase (HMGCR) and squalene mono-oxygenase being the rate-limiting enzymes. Cholesterol synthesis is tightly regulated, encompassing both transcriptional and post-transcriptional regulators and feedback mechanisms [39]. If critical illness would reduce cholesterol synthesis, this would, in a context of reduced intake, potentially lead to lower circulating cholesterol levels. Patients with liver failure are often presented with decreased serum cholesterol levels related to a reduced HDL-cholesterol and apolipoprotein synthesis [13]. In addition, a decrease in the cholesterol precursors squalene and lathosterol was observed in trauma ICU patients, indeed suggesting reduced cholesterol synthesis [11, 15]. However, a study performed in septic rats reported an elevated rather than suppressed hepatic cholesterol synthesis [40].

#### Cholesterol metabolism and conversion during critical illness

Other factors such as loss of lipoproteins, hemodilution or an accelerated metabolism might also contribute to an altered cholesterol availability in critically ill patients [41, 42]. The liver is the main cholesterol metabolizing organ in the body (Fig. 1). After peripheral uptake of cholesterol in cells, excess cholesterol is removed from peripheral tissues to the liver in the process of reverse cholesterol transport for further metabolism or excretion from the body via the bile [39]. Where only a small fraction of bile acids is excreted in the feces, the majority returns to the liver via the enterohepatic cycle. The enterohepatic cycle is however often disturbed during critical illness, due to a reduced enteral intake, gut impairment, diarrhea and cholestasis [43, 44]. Indeed, critically ill patients in the protracted phase of illness often display elevated bile acid concentrations [45]. These cholestatic features have been attributed to ongoing bile acid synthesis with loss of feedback inhibition and alterations in transport and conjugation [45], suggesting an increased conversion from cholesterol to bile acids. Interestingly, the decrease in plasma cholesterol observed after surgery, trauma or sepsis is somewhat attenuated by the presence of cholestasis [11, 13]. Furthermore, pro-inflammatory cytokines can increase the activity of cholesterol 25-hydroxylase and the acute phase protein phospholipase A2, thereby also affecting metabolism of apolipoproteins and cholesterol esters [46].

An increased conversion of cholesterol to steroid hormones might theoretically also be involved as cholesterol is required for the sustained conversion to cortisol in the adrenal cortex as part of the stress response to critical illness [31]. The sicker the patient, the higher plasma cortisol concentrations are and the lower plasma cholesterol concentrations. Other cholesterol-derived metabolites, such as aldosterone, sex hormones and vitamin D might also be involved but circulating sex hormones and vitamin D are decreased, and not elevated during critical illness [47]. Tracer data on the distribution and conversion of cholesterol to cholesterol-derived metabolites during critical illness is required to further clarify the involvement of cholesterol metabolism and conversion to the hypocholesterolemia of critical illness. Importantly, the adrenal cortisol response to ACTH correlated with HDL-cholesterol concentrations in critically ill patients [48] and patients suffering from prolonged critical illness demonstrated cholesterol-depleted adrenal glands [49]. Whether the sustained hypocholesterolemia is involved in failing adrenal function in protracted critical illness [31] needs to be further investigated.

### Therapeutic implications

#### Statins

Patients at risk of atherosclerotic cardiovascular disease can benefit from lipid-lowering drugs such as statins. Statins are most commonly used to lower cholesterol concentrations by their direct action on HMGCR (Fig. 1) but also have anti-inflammatory and immunomodulatory properties, which theoretically may help mitigate the inflammatory response and improve outcome during critical illness [50]. Meta-analyses of randomized controlled trials (RCTs) and observational studies on statin use in ICU patients had limited power to study hard clinical endpoints, did not improve mortality in patients with sepsis and argue against their use during critical illness (reviewed in [51]). Furthermore, statin treatment is associated with muscle toxicity and myopathy [52]. Lower serum cholesterol levels have been recently documented in critically ill patients suffering from ICUAW as compared with non-weak patients [20]. Whether a beneficial effect of statins on inflammation, immunity or on endothelial function was outweighed by a suppressive effect on cholesterol availability cannot be concluded from these studies. In conclusion, current evidence argues against the use of statins in the management of critical illness. A close monitoring of high-risk patients already taking lipid-lowering drugs might be necessary to adapt the dose regimen, as an abrupt withdrawal in may cause negative inflammatory rebound effects, as was demonstrated in a myocardial infarction population [51, 53].

#### Substitution therapy?

Although there is a clear invert association between plasma cholesterol and mortality in septic and other critically ill patients, intervention studies to investigate causality are not (yet) available. Therapies mimicking the endotoxin scavenging mechanisms of cholesterol, such as treatment with a phospholipid emulsion or polymyxin B hemoperfusion were unsuccessful in improving outcome [54].

A novel antitoxin liposomal agent, CAL02, which is a cholesterol-containing liposomal preparation, has been tested in severe pneumococcal pneumonia for safety but not yet for efficacy [55]. A phase I/II feasibility trial is ongoing to test whether a lipid emulsion can stabilize cholesterol levels in septic patients, but no clinical endpoints will be investigated [56]. In preclinical studies, infusion with reconstituted HDL or apolipoprotein A1 improved organ function and survival in rodent models of sepsis and endotoxemia [57–59]. Pharmacological inhibition of CETP with anacetrapib preserved HDL-cholesterol and apolipoprotein A1 levels and increased survival in septic mice [38]. Its therapeutic potential is also strengthened by the observation that a CETP gain-of-function genetic variant is associated with increased sepsis mortality [38].

In conclusion, large RCTs on cholesterol and/or lipoprotein supplementation demonstrating safety, tolerability, and efficacy are currently lacking, also prevailing any conclusion on whether hypocholesterolemia should be rather interpreted as a good clinical marker of severity of illness or warrants treatment.

## Fatty acid and triglyceride homeostasis during critical illness

### The role of fatty acids and triglycerides during critical illness

During the acute and subacute phase of critical illness, FFA and, less frequently observed, triglycerides appear increased [16, 17]. The acute increase of FFA is more pronounced with sepsis or septic shock [16] and is higher in non-survivors compared to survivors [7]. Elevated serum triglyceride concentrations show a linear positive association with increased mortality [8] and have been described to reflect the severity of critical illness [9]. In contrast to the observed association of infection or sepsis with low plasma cholesterol [10, 11, 15] and high FFA [16], plasma triglyceride levels are not found different between infectious or non-infectious patients [60].

**Fig. 2:**
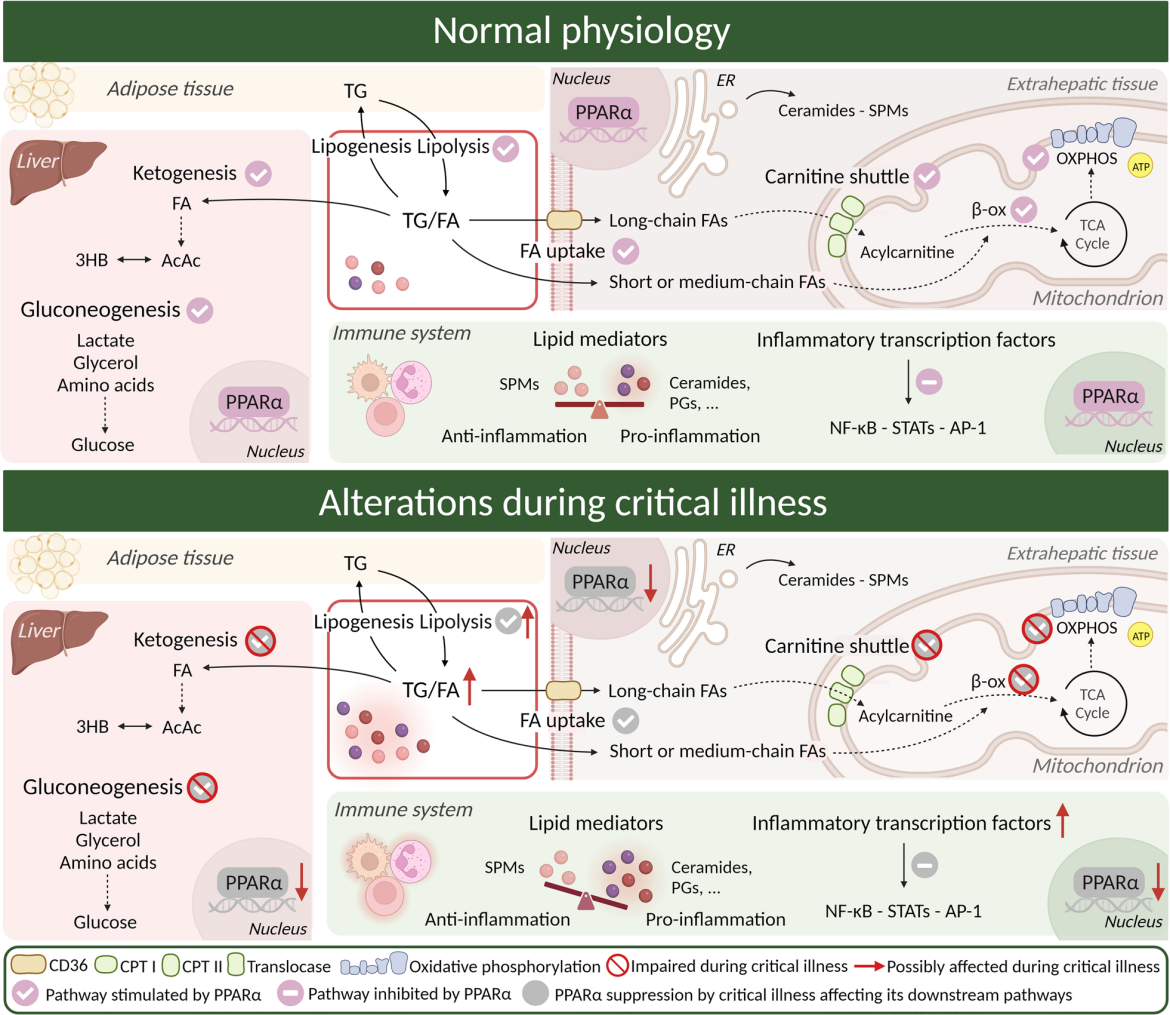
Schematic overview of normal lipid physiology and possible mechanisms involved in lipid disturbances during critical illness. In normal physiology, circulating fatty acid concentration depends on the balance of lipolysis and lipogenesis. Fatty acid uptake is mediated by transporters and passive diffusion and will either enter oxidative pathways to provide ATP or converted to ketone bodies, or stored as triglycerides. The nuclear receptor PPARα is the key transcriptional regulator of these processes. Fatty acids can be converted to immunomodulatory mediators such as ceramides, prostaglandins and specialized pro-resolving mediators. During critical illness, circulating fatty acids and triglycerides are increased and lipid mediators are imbalanced to a pro-inflammatory shift. Elevated lipolysis, impaired oxidative processes and hampered ketogenesis are observed in a context of suppressed PPARα expression. TG: triglyceride; FA; fatty acid; 3HB: beta-hydroxybutyrate; AcAc: acetoacetate; SPMs: specialized pro-resolving mediators; PPARα: peroxisome-proliferator-activated receptor α; ER: endoplasmic reticulum; β-ox: beta-oxidation; TCA cycle: tricarboxylic acid cycle; ATP: adenosine triphosphate; CPT: carnitine palmitoyltransferase; OXPHOS: oxidative phosphorylation; PGs: prostaglandins; NF-κB: Nuclear factor kappa-light-chain-enhancer of activated B cells; STATs: signal transducer of activation (STAT) proteins; AP-1: Activator protein 1; CD36: cluster of differentiation 36. Created with Biorender.com

Increased circulating lipids may have broad metabolic and inflammatory implications (Fig. 2). They do not only comprise energy-dense compounds, but may also yield a multitude of inflammatory mediators [61]. These mediators may promote inflammation (e.g., eicosanoids, prostaglandins and leukotrienes), indispensable during the acute phase of critical illness, or attenuate the immune response (e.g., specialized pro-resolving mediators (SPMs)), by restoring homeostasis and enhancing recovery processes [61]. As such, a dysregulated lipid balance may affect both survival during the acute phase of critical illness, and may contribute to the unabated and detrimental inflammation observed in chronic critically ill patients.

### Pathophysiology

#### Altered transport and uptake of fatty acids

The delivery and uptake of FFA and triglycerides was originally conceptualized as a process of passive diffusion, but recent evidence indicates that cellular and mitochondrial uptake of long-chain FA (LCFA) is a tightly regulated process (Fig. 2) [62]. FFAs are first dissociated from albumin or liberated from lipoproteins by lipoprotein lipase, and afterwards taken up by a complex array of proteins, among which the receptor cluster of differentiation 36 (CD36) is one of the most extensively researched [63]. The observed hypertriglyceridemia during critical illness may indirectly indicate decreased cellular uptake, but post-mortem biopsies from adipose tissue indicated increased uptake of FFAs [16, 17, 64]. Additionally, the rate-limiting enzyme of intramitochondrial LCFA transport, carnitine palmitoyl transferase I (CPT1), was suppressed in critically ill animal models in liver and heart tissues [65].

Carnitine may bind LCFA to facilitate intramitochondrial transport, but may also maintain mitochondrial coenzyme A pools by scavenging fatty acyl intermediates [66]. A deficiency in carnitine and its acyl-derivates may as such reflect impaired lipid oxidation and mitochondrial dysfunction [66]. Metabolomic studies in septic patients and patients with respiratory failure have shown great disparity in acylcarnitine plasma profile between survivors and non-survivors [67, 68]. Whether circulating acylcarnitine metabolites may be useful as biomarkers of disturbed cellular mitochondrial integrity and metabolic capacity, needs further investigation.

#### Changes in lipolysis, lipogenesis and lipid oxidation

Sepsis and other critical illnesses evoke an acute stress response with increased catecholamines, glucagon, growth hormone and cortisol plasma levels and induce relative insulin resistance, provoking a lipolytic response by stimulating lipase activity [47, 69]. The increase of FFA might as such simply reflect the severity of illness and its evoked acute stress response. Indeed, increased lipolysis is one of the common metabolic signals of acute critical illness and may be more pronounced in patients with shock [16, 17]. Nevertheless, the improved outcome observed in obese patients in the ICU (obesity paradox, vide infra) appears to be mediated among others by more efficient lipolytic processes [70].

The role of lipogenesis in critically ill patients remains uncertain. Lipogenesis may provide a protective cellular response to alleviate lipotoxicity by sequestering circulating lipids and glucose especially in the context of the stress-induced hyperglycemia, which is associated with increased mortality [4, 5]. Indeed, post-mortem biopsies from adipose tissues of critically ill patients revealed that lipid synthesis and glucose uptake appeared increased [64]. Contrary, cellular dysfunction may impair the oxidation of metabolic substrates which may result in citrate accumulating by an overflowing tricarboxylic acid (TCA) cycle, especially in the presence of (high doses of) nutrients [66]. Excess citrate may increase malonyl-CoA (the rate-limiting metabolite of lipogenesis) and thus activate inappropriate lipogenesis [66]. Inappropriate lipogenesis may waste both precious energy molecules as an energy consuming process and promote harmful lipid accumulation in tissues. As such the precise relation between lipolysis and lipogenesis in different stage of the disease process remains unclear and may be beneficial or harmful depending on the origin or context (Fig. 2).

As beta-oxidation and mitochondrial function become impaired during critical illness, metabolic pathways shift towards glycolysis [71]. This shift has mostly been described in immune cells, and may reflect adaptive mechanisms to stimulate defensive processes (e.g., immune response) [71]. Concurrent cellular processes may be diminished to spare and reprioritize energy towards vital functions (metabolic tolerance) [71]. Restoration of lipid and mitochondrial oxidative pathways is, however, essential in order for recovery to occur. Proteomics and metabolomics studies in critically ill patients illustrated that non-surviving patients had defective lipid oxidative pathways and impaired intramitochondrial lipid transport [68]. On a cellular level, transcription of lipid transport and oxidation is regulated by nuclear receptor peroxisome-proliferator receptor alpha (PPARα). PPARα mediates the switch from glucose to lipid oxidation, activates ketogenesis but also mediates anti-inflammatory actions (Fig. 2). PPARα expression was decreased in post-mortem biopsies of critically ill patients and this downregulation was shown to correlate with sepsis severity [72, 73]. Moreover, PPARα activation has been proposed to have beneficial effects after (major) trauma, traumatic brain injury and spinal injury [74].

Obese patients comprise a unique population within the ICU due to their preexisting dyslipidemia and altered lipid and cholesterol metabolism. Remarkably, several large cohort studies and meta-analyses have described a lower mortality risk for critically ill patients with excess body fat [75, 76]. Human obese patients displayed lower plasma levels of inflammatory cytokines when compared with lean patients and had a lower incidence of ICUAW [70, 77]. Furthermore, obese patients also displayed reduced protein catabolism compared with lean patients [78]. In septic mice, the improved muscle function was found to be related to an increased mobilization of fat from adipose tissue, and subsequent increased hepatic FA oxidation and ketone formation [79]. This suggests that obese ICU patients might have a primed metabolic profile which favors the release and the use of stored energy from adipose tissue. Together, these may counteract the use of ectopic lipids and proteins from vital organs and muscle, hence averting lean tissue wasting. Whether the increased rate of lipolysis and ketogenesis contributes to the improved survival in critical illness, or whether it also has maladaptive consequences needs further investigation.

#### Regulation of bioactive lipid mediators

The immune response to critical illness appears to involve a complex dysregulation of bioactive lipid mediators. Sepsis non-survivors were characterized by a distinct profile of lipid mediators with derangements in both pro-resolving mediators and pro-inflammatory metabolites [80]. Polyunsaturated fatty acids (PUFAs) are the primary precursors of inflammatory lipid mediators. Among these, arachidonic acid-derived metabolites of omega-6 PUFAs promote inflammation, whereas eicosapentaenoic and docosahexaenoic acid (EPA/DHA) omega-3 PUFAs are considered more anti-inflammatory. These anti-inflammatory actions may arise by SPMs (resolvins, protectin, maresins and lipoxins) that are primarily derived from omega-3 FAs [81]. SPMs facilitate resolution of inflammation, tissue regeneration and pathogen clearance and may play a crucial role in preventing the inappropriate escalation of the immune response both in the acute and chronic phase of critical illness. Supplementation of SPMs in animal models of sepsis improved mortality, decreased oxidative stress and attenuated inflammation [82, 83] and trauma patients with uncomplicated recovery had a higher expression of resolvins than patients with a complicated recovery [84]. In addition to SPMs, ceramides may affect immune dysregulation during critical illness. Ceramides are a type of sphingolipids with primary signaling functions that play a central role during intracellular stress and regulation of apoptosis [85]. Observational research in critically ill patients has shown an association between increased ceramide concentrations and poor survival in septic patients [86]. Although specific lipid mediators might pose interesting new strategies to predict outcomes and adapt therapy, this topic is beyond the scope of this article and has been extensively reviewed elsewhere [61, 81].

### Therapeutic targets

#### Parenteral nutrition strategies

Lipids in enteral and parenteral nutrition function as a source of energy-dense calories, sparing carbohydrate requirements, and provide essential FAs that are indispensable for cell membrane structure and function. Modern lipid mixtures may, however, also modulate the inflammatory response and metabolic functioning according to FA chain length and triglyceride structure. The immunomodulatory properties of PUFAs have received considerable attention in the field of immunonutrition. As such, traditional soy bean oils have become scrutinized as a result of their pro-inflammatory and immunosuppressive effects in comparison to other lipid emulsions [33]. These side-effects have mostly been attributed to the high ratio of omega-6 PUFAs present in soybean oil, while lipid emulsions rich in omega-3 PUFAs (fish oil) and omega-9 PUFAs (olive oil) are considered anti-inflammatory or neutral, respectively [33, 61]. Although few smaller RCTs in critically ill patients suggest a decrease in the hospital length of stay and rate of infections after infusion of omega-3 FA rich lipid emulsion, meta-analyses have generated inconclusive results [87, 88].

Besides immunomodulation, lipid emulsions may also alleviate metabolic dysfunction during critical illness. Mixtures rich in medium-chain triglycerides (MCTs) elicit a stronger ketogenic response than emulsions wit long-chain triglycerides [89]. Ketone bodies may serve as alternative energy substrates to carbohydrates and may activate beneficial signaling cascades that enhance resilience to oxidative stress and promote recovery [90, 91]. High-quality RCTs should assess whether the protective effects on mitochondrial function and muscle integrity now only observed in small RCTs, might result in robust clinical benefit [92–94]. Alternatively, (relative) macronutrient restriction may also promote favorable ketone body and lipid oxidative processes. A secondary analysis of a pediatric nutritional RCT revealed that plasma ketosis statistically mediated the benefits of withholding parenteral nutrition in the first week of critical illness [91].

#### Targeting metabolic pathways

Pharmacological PPARα activation might overcome its downregulation and subsequent compromised downstream pathways during critical illness. Although fibrates, PPARα agonists, improve dyslipidemia but not mortality in patients with cardiovascular risk factors, clinical trials in critically ill patients are sparse [95]. A few smaller studies in children with burn wounds showed that pharmacological activation of PPARα improved mitochondrial lipid oxidation [96, 97].

Critically ill patients may be prone to develop (relative) carnitine deficiency as renal losses are increased, endogenous production may be hampered, and nutritional intake is diminished [66]. Carnitine deficiency may impair intramitochondrial transport of LCFA and disturb coenzyme A homeostasis [66]. A small RCT found a slightly lower mortality after carnitine infusion in septic shock patients [98]. Carnitine supplementation improved inflammatory markers in critically ill patients and had a small effect in patients with septic shock, but these findings were not reproduced in a phase 2 study [98–100]. These trials were, however, conducted irrespective of carnitine status by including a population not requiring exogenous supplementation. As such, carnitine supplementation in (relative) carnitine deficient patients might still potentially optimize metabolic pathways and enforce clinical effects, but more research on this topic is required.

#### Hypertriglyceridemia induced by critical care-related therapies

Hypertriglyceridemia by (over)feeding or defective oxidative pathways may induce lipotoxicity [72, 101]. Especially in patients with concomitant hyperglycemia, intensive insulin therapy may attenuate circulating triglycerides levels, which statistically mediates part of the outcome benefit of tight glycemic control [8]. In contrast, propofol, a sedative-hypnotic medication, is dissolved in a lipid emulsion and may even provoke hypertriglyceridemia, especially after prolonged administration [102]. In extreme cases, propofol and its lipid carrier may overwhelm mitochondrial oxidation, designated the propofol infusion syndrome. Although interruption of this anesthetic drug may be sufficient in an early phase, death may be inevitable once metabolic disruption and mitochondrial uncoupling has set fort [103].

## Conclusion

Critical illness induces significant perturbations in lipid and cholesterol homeostasis and is characterized by low total-, LDL- and HDL-cholesterol plasma concentrations, together with a, less pronounced, increase in plasma FFA. Hypocholesterolemia of critical illness is strongly associated with severity of illness, and is considered to be part of the acute phase response. Reduced nutritional uptake, increased scavenging of lipoproteins as well as an increased conversion to cortisol or other cholesterol-derived metabolites might all play a role in the decrease in plasma cholesterol. One could speculate that sustained low cholesterol concentrations might become disadvantageous in the prolonged phase of critical illness, because of a diminished responsiveness to tissue stress and a reduced delivery to liver and steroidogenic tissues. Remarkably, a reduced mortality risk was observed in critically ill obese patients who typically display a metabolic profile with dyslipidemia [75, 76]. The acute stress response to critical illness creates a lipolytic cocktail, which might explain the increase in plasma FFA, although reduced uptake and oxidation of FFA, but also increased lipogenesis, especially in prolonged critical illness, will also affect the circulating levels. Whether the increase in lipids can be considered adaptive, as necessary energy substrate or essential components for cellular function, or might also have detrimental lipotoxic consequences should be further investigated.

## Data Availability

Not applicable as no datasets were used for this review.

## References

[1] Teblick A, Gunst J, Langouche L, Van den Berghe G (2022). Novel insights in endocrine and metabolic pathways in sepsis and gaps for future research. Clin Sci.

[2] Vanhorebeek I, Latronico N, Van den Berghe G (2020). ICU-acquired weakness. Intensive Care Med.

[3] Gunst J, Casaer MP, Preiser JC, Reignier J, Van den Berghe G (2023). Toward nutrition improving outcome of critically ill patients: how to interpret recent feeding RCTs?. Crit Care.

[4] Gunst J, De Bruyn A, Van den Berghe G (2019). Glucose control in the ICU. Curr Opin Anaesthesiol.

[5] Preiser JC, van Zanten AR, Berger MM, Biolo G, Casaer MP, Doig GS, Griffiths RD, Heyland DK, Hiesmayr M, Iapichino G (2015). Metabolic and nutritional support of critically ill patients: consensus and controversies. Crit Care.

[6] Hofmaenner DA, Arina P, Kleyman A, Page Black L, Salomao R, Tanaka S, Guirgis FW, Arulkumaran N, Singer M (2023). Association between hypocholesterolemia and mortality in critically ill patients with sepsis: a systematic review and meta-analysis. Crit Care Explor.

[7] Nogueira AC, Kawabata V, Biselli P, Lins MH, Valeri C, Seckler M, Hoshino W, Junior LG, Bernik MM, de Andrade Machado JB (2008). Changes in plasma free fatty acid levels in septic patients are associated with cardiac damage and reduction in heart rate variability. Shock.

[8] Mesotten D, Swinnen JV, Vanderhoydonc F, Wouters PJ, Van den Berghe G (2004). Contribution of circulating lipids to the improved outcome of critical illness by glycemic control with intensive insulin therapy. J Clin Endocrinol Metab.

[9] Lind L, Lithell H (1994). Impaired glucose and lipid metabolism seen in intensive care patients is related to severity of illness and survival. Clin Intensive Care.

[10] Tanaka S, Labreuche J, Drumez E, Harrois A, Hamada S, Vigue B, Couret D, Duranteau J, Meilhac O (2017). Low HDL levels in sepsis versus trauma patients in intensive care unit. Ann Intensive Care.

[11] Chiarla C, Giovannini I, Giuliante F, Zadak Z, Vellone M, Ardito F, Clemente G, Murazio M, Nuzzo G (2010). Severe hypocholesterolemia in surgical patients, sepsis, and critical illness. J Crit Care.

[12] Coombes EJ, Shakespeare PG, Batstone GF (1980). Lipoprotein changes after burn injury in man. J Trauma.

[13] Giovannini I, Boldrini G, Chiarla C, Giuliante F, Vellone M, Nuzzo G (1999). Pathophysiologic correlates of hypocholesterolemia in critically ill surgical patients. Intensive Care Med.

[14] Gordon BR, Parker TS, Levine DM, Saal SD, Wang JC, Sloan BJ, Barie PS, Rubin AL (2001). Relationship of hypolipidemia to cytokine concentrations and outcomes in critically ill surgical patients. Crit Care Med.

[15] Bakalar B, Hyspler R, Pachl J, Zadak Z (2003). Changes in cholesterol and its precursors during the first days after major trauma. Wien Klin Wochenschr.

[16] Ilias I, Vassiliadi DA, Theodorakopoulou M, Boutati E, Maratou E, Mitrou P, Nikitas N, Apollonatou S, Dimitriadis G, Armaganidis A (2014). Adipose tissue lipolysis and circulating lipids in acute and subacute critical illness: effects of shock and treatment. J Crit Care.

[17] Dimopoulou I, Nikitas N, Orfanos SE, Theodorakopoulou M, Vassiliadi D, Ilias I, Ikonomidis I, Boutati E, Maratou E, Tsangaris I (2011). Kinetics of adipose tissue microdialysis-derived metabolites in critically ill septic patients: associations with sepsis severity and clinical outcome. Shock.

[18] Dunham CM, Fealk MH, Sever WE (2003). Following severe injury, hypocholesterolemia improves with convalescence but persists with organ failure or onset of infection. Crit Care.

[19] van Leeuwen HJ, Heezius EC, Dallinga GM, van Strijp JA, Verhoef J, van Kessel KP (2003). Lipoprotein metabolism in patients with severe sepsis. Crit Care Med.

[20] Goossens C, Weckx R, Derde S, Vander Perre S, Derese I, Van Veldhoven PP, Ghesquiere B, Van den Berghe G, Langouche L (2021). Altered cholesterol homeostasis in critical illness-induced muscle weakness: effect of exogenous 3-hydroxybutyrate. Crit Care.

[21] Jafurulla M, Chattopadhyay A (2013). Membrane lipids in the function of serotonin and adrenergic receptors. Curr Med Chem.

[22] Dunser MW, Ruokonen E, Pettila V, Ulmer H, Torgersen C, Schmittinger CA, Jakob S, Takala J (2009). Association of arterial blood pressure and vasopressor load with septic shock mortality: a post hoc analysis of a multicenter trial. Crit Care.

[23] Levels JH, Abraham PR, van den Ende A, van Deventer SJ (2001). Distribution and kinetics of lipoprotein-bound endotoxin. Infect Immun.

[24] Parker TS, Levine DM, Chang JC, Laxer J, Coffin CC, Rubin AL (1995). Reconstituted high-density lipoprotein neutralizes gram-negative bacterial lipopolysaccharides in human whole blood. Infect Immun.

[25] Vreugdenhil AC, Snoek AM, van’t Veer C, Greve JW, Buurman WA (2001). LPS-binding protein circulates in association with apoB-containing lipoproteins and enhances endotoxin-LDL/VLDL interaction. J Clin Invest.

[26] Kallio KA, Buhlin K, Jauhiainen M, Keva R, Tuomainen AM, Klinge B, Gustafsson A, Pussinen PJ (2008). Lipopolysaccharide associates with pro-atherogenic lipoproteins in periodontitis patients. Innate Immun.

[27] Yu B, Hailman E, Wright SD (1997). Lipopolysaccharide binding protein and soluble CD14 catalyze exchange of phospholipids. J Clin Invest.

[28] Vitols S, Gahrton G, Bjorkholm M, Peterson C (1985). Hypocholesterolaemia in malignancy due to elevated low-density-lipoprotein-receptor activity in tumour cells: evidence from studies in patients with leukaemia. Lancet.

[29] Tan JT, Prosser HC, Dunn LL, Vanags LZ, Ridiandries A, Tsatralis T, Lecce L, Clayton ZE, Yuen SC, Robertson S (2016). High-density lipoproteins rescue diabetes-impaired angiogenesis via scavenger receptor class B type I. Diabetes.

[30] Tsatralis T, Ridiandries A, Robertson S, Vanags LZ, Lam YT, Tan JT, Ng MK, Bursill CA (2016). Reconstituted high-density lipoproteins promote wound repair and blood flow recovery in response to ischemia in aged mice. Lipids Health Dis.

[31] Peeters B, Meersseman P, Vander Perre S, Wouters PJ, Vanmarcke D, Debaveye Y, Billen J, Vermeersch P, Langouche L, Van den Berghe G (2018). Adrenocortical function during prolonged critical illness and beyond: a prospective observational study. Intensive Care Med.

[32] Muscaritoli M, Pradelli L (2021). Medium-chain triglyceride (MCT) content of adult enteral tube feeding formulas and clinical outcomes. A systematic review. Front Nutr.

[33] Calder PC, Adolph M, Deutz NE, Grau T, Innes JK, Klek S, Lev S, Mayer K, Michael-Titus AT, Pradelli L (2018). Lipids in the intensive care unit: recommendations from the ESPEN Expert Group. Clin Nutr.

[34] Ali Abdelhamid Y, Cousins CE, Sim JA, Bellon MS, Nguyen NQ, Horowitz M, Chapman MJ, Deane AM (2015). Effect of critical illness on triglyceride absorption. JPEN J Parenter Enteral Nutr.

[35] Levels JHM, Pajkrt D, Schultz M, Hoek FJ, van Tol A, Meijers JCM, van Deventer SJH (2007). Alterations in lipoprotein homeostasis during human experimental endotoxemia and clinical sepsis. Bba-Mol Cell Biol L.

[36] de la Llera MM, McGillicuddy FC, Hinkle CC, Byrne M, Joshi MR, Nguyen V, Tabita-Martinez J, Wolfe ML, Badellino K, Pruscino L (2012). Inflammation modulates human HDL composition and function in vivo. Atherosclerosis.

[37] Masucci-Magoulas L, Moulin P, Jiang XC, Richardson H, Walsh A, Breslow JL, Tall A (1995). Decreased cholesteryl ester transfer protein (CETP) mRNA and protein and increased high density lipoprotein following lipopolysaccharide administration in human CETP transgenic mice. J Clin Invest.

[38] Trinder M, Wang Y, Madsen CM, Ponomarev T, Bohunek L, Daisely BA, Julia Kong H, Blauw LL, Nordestgaard BG, Tybjaerg-Hansen A (2021). Inhibition of cholesteryl ester transfer protein preserves high-density lipoprotein cholesterol and improves survival in sepsis. Circulation.

[39] Luo J, Yang H, Song BL (2020). Mechanisms and regulation of cholesterol homeostasis. Nat Rev Mol Cell Biol.

[40] de Vasconcelos PR, Kettlewell MG, Gibbons GF, Williamson DH (1989). Increased rates of hepatic cholesterogenesis and fatty acid synthesis in septic rats in vivo: evidence for the possible involvement of insulin. Clin Sci (Lond).

[41] Sun X, Oberlander D, Huang J, Weissman C (1998). Fluid resuscitation, nutritional support, and cholesterol in critically ill postsurgical patients. J Clin Anesth.

[42] Memis D, Gursoy O, Tasdogan M, Sut N, Kurt I, Ture M, Karamanlioglu B (2007). High C-reactive protein and low cholesterol levels are prognostic markers of survival in severe sepsis. J Clin Anesth.

[43] Yoseph BP, Klingensmith NJ, Liang Z, Breed ER, Burd EM, Mittal R, Dominguez JA, Petrie B, Ford ML, Coopersmith CM (2016). Mechanisms of intestinal barrier dysfunction in sepsis. Shock.

[44] Kiss O, Maizik J, Tamme K, Orav A, van de Poll MCG, Reintam Blaser A (2020). Diarrhea and elevation of plasma markers of cholestasis are common and often occur concomitantly in critically ill patients. J Crit Care.

[45] Jenniskens M, Langouche L, Van den Berghe G (2018). Cholestatic alterations in the critically ill: some new light on an old problem. Chest.

[46] Diczfalusy U, Olofsson KE, Carlsson AM, Gong M, Golenbock DT, Rooyackers O, Flaring U, Bjorkbacka H (2009). Marked upregulation of cholesterol 25-hydroxylase expression by lipopolysaccharide. J Lipid Res.

[47] Teblick A, Langouche L, Van den Berghe G (2019). Anterior pituitary function in critical illness. Endocr Connect.

[48] van der Voort PHJ, Gerritsen RT, Bakker AJ, Boerma EC, Kuiper MA, de Heide L (2003). HDL-cholesterol level and cortisol response to synacthen in critically ill patients. Intensive Care Med.

[49] Boonen E, Langouche L, Janssens T, Meersseman P, Vervenne H, De Samblanx E, Pironet Z, Van Dyck L, Vander Perre S, Derese I (2014). Impact of duration of critical illness on the adrenal glands of human intensive care patients. J Clin Endocrinol Metab.

[50] Mekontso-Dessap A, Brun-Buisson C (2006). Statins: the next step in adjuvant therapy for sepsis?. Intensive Care Med.

[51] Al-Husinat L, Abu Hmaid A, Abbas H, Abuelsamen B, Albelbisi M, Haddad S, Qamileh I, Quneis O, Al Modanat ZJ, Ferrara G (2023). Role of aspirin, beta-blocker, statins, and heparin therapy in septic patients under mechanical ventilation: a narrative review. Front Med.

[52] Nguyen KA, Li L, Lu D, Yazdanparast A, Wang L, Kreutz RP, Whipple EC, Schleyer TK (2018). A comprehensive review and meta-analysis of risk factors for statin-induced myopathy. Eur J Clin Pharmacol.

[53] Sposito AC, Carvalho LS, Cintra RM, Araujo AL, Ono AH, Andrade JM, Coelho OR, Quinaglia e Silva JC, Brasilia Heart Study G (2009). Rebound inflammatory response during the acute phase of myocardial infarction after simvastatin withdrawal. Atherosclerosis.

[54] Dellinger RP, Bagshaw SM, Antonelli M, Foster DM, Klein DJ, Marshall JC, Palevsky PM, Weisberg LS, Schorr CA, Trzeciak S (2018). Effect of targeted polymyxin B hemoperfusion on 28-day mortality in patients with septic shock and elevated endotoxin level: the EUPHRATES Randomized Clinical Trial. JAMA.

[55] Laterre PF, Colin G, Dequin PF, Dugernier T, Boulain T, Azeredo da Silveira S, Lajaunias F, Perez A, Francois B (2019). CAL02, a novel antitoxin liposomal agent, in severe pneumococcal pneumonia: a first-in-human, double-blind, placebo-controlled, randomised trial. Lancet Infect Dis.

[56] Guirgis FW, Black LP, Rosenthal MD, Henson M, Ferreira J, Leeuwenburgh C, Kalynych C, Moldawer LL, Miller T, Jones L (2019). LIPid Intensive Drug therapy for Sepsis Pilot (LIPIDS-P): Phase I/II clinical trial protocol of lipid emulsion therapy for stabilising cholesterol levels in sepsis and septic shock. BMJ Open.

[57] Moreira RS, Irigoyen M, Sanches TR, Volpini RA, Camara NO, Malheiros DM, Shimizu MH, Seguro AC, Andrade L (2014). Apolipoprotein A-I mimetic peptide 4F attenuates kidney injury, heart injury, and endothelial dysfunction in sepsis. Am J Physiol Regul Integr Comp Physiol.

[58] McDonald MC, Dhadly P, Cockerill GW, Cuzzocrea S, Mota-Filipe H, Hinds CJ, Miller NE, Thiemermann C (2003). Reconstituted high-density lipoprotein attenuates organ injury and adhesion molecule expression in a rodent model of endotoxic shock. Shock.

[59] Tanaka S, Geneve C, Zappella N, Yong-Sang J, Planesse C, Louedec L, Viranaicken W, Bringart M, Montravers P, Denamur E (2020). Reconstituted high-density lipoprotein therapy improves survival in mouse models of sepsis. Anesthesiology.

[60] Luthold S, Berneis K, Bady P, Muller B (2007). Effects of infectious disease on plasma lipids and their diagnostic significance in critical illness. Eur J Clin Invest.

[61] Singer P, Shapiro H, Theilla M, Anbar R, Singer J, Cohen J (2008). Anti-inflammatory properties of omega-3 fatty acids in critical illness: novel mechanisms and an integrative perspective. Intensive Care Med.

[62] Ehehalt R, Füllekrug J, Pohl J, Ring A, Herrmann T, Stremmel W (2006). Translocation of long chain fatty acids across the plasma membrane–lipid rafts and fatty acid transport proteins. Mol Cell Biochem.

[63] Muniz-Santos R, Lucieri-Costa G, de Almeida MAP, Moraes-de-Souza I, Brito M, Silva AR, Goncalves-de-Albuquerque CF (2023). Lipid oxidation dysregulation: an emerging player in the pathophysiology of sepsis. Front Immunol.

[64] Langouche L, Perre SV, Thiessen S, Gunst J, Hermans G, D'Hoore A, Kola B, Korbonits M, Van den Berghe G (2010). Alterations in adipose tissue during critical illness: an adaptive and protective response?. Am J Respir Crit Care Med.

[65] Takeyama N, Itoh Y, Kitazawa Y, Tanaka T (1990). Altered hepatic mitochondrial fatty acid oxidation and ketogenesis in endotoxic rats. Am J Physiol.

[66] Bonafe L, Berger MM, Que YA, Mechanick JI (2014). Carnitine deficiency in chronic critical illness. Curr Opin Clin Nutr Metab Care.

[67] Rousseau AF, Schmitz S, Cavalier E, Misset B, Boemer F (2022). Altered serum acylcarnitines profile after a prolonged stay in intensive care. Nutrients.

[68] Langley RJ, Tsalik EL, van Velkinburgh JC, Glickman SW, Rice BJ, Wang C, Chen B, Carin L, Suarez A, Mohney RP (2013). An integrated clinico-metabolomic model improves prediction of death in sepsis. Sci Transl Med.

[69] Choi SM, Tucker DF, Gross DN, Easton RM, DiPilato LM, Dean AS, Monks BR, Birnbaum MJ (2010). Insulin regulates adipocyte lipolysis via an Akt-independent signaling pathway. Mol Cell Biol.

[70] Goossens C, Marques MB, Derde S, Vander Perre S, Dufour T, Thiessen SE, Guiza F, Janssens T, Hermans G, Vanhorebeek I (2017). Premorbid obesity, but not nutrition, prevents critical illness-induced muscle wasting and weakness. J Cachexia Sarcopenia Muscle.

[71] Liu J, Zhou G, Wang X, Liu D (2022). Metabolic reprogramming consequences of sepsis: adaptations and contradictions. Cell Mol Life Sci.

[72] Paumelle R, Haas JT, Hennuyer N, Bauge E, Deleye Y, Mesotten D, Langouche L, Vanhoutte J, Cudejko C, Wouters K (2019). Hepatic PPARalpha is critical in the metabolic adaptation to sepsis. J Hepatol.

[73] Standage SW, Caldwell CC, Zingarelli B, Wong HR (2012). Reduced peroxisome proliferator-activated receptor α expression is associated with decreased survival and increased tissue bacterial load in sepsis. Shock.

[74] Neher MD, Weckbach S, Huber-Lang MS, Stahel PF (2012). New insights into the role of peroxisome proliferator-activated receptors in regulating the inflammatory response after tissue injury. PPAR Res.

[75] Pepper DJ, Demirkale CY, Sun J, Rhee C, Fram D, Eichacker P, Klompas M, Suffredini AF, Kadri SS (2019). Does obesity protect against death in sepsis? A retrospective cohort study of 55,038 adult patients. Crit Care Med.

[76] Sakr Y, Alhussami I, Nanchal R, Wunderink RG, Pellis T, Wittebole X, Martin-Loeches I, Francois B, Leone M, Vincent JL (2015). Being overweight is associated with greater survival in ICU patients: results from the intensive care over nations audit. Crit Care Med.

[77] Wacharasint P, Boyd JH, Russell JA, Walley KR (2013). One size does not fit all in severe infection: obesity alters outcome, susceptibility, treatment, and inflammatory response. Crit Care.

[78] Vankrunkelsven W, Derde S, Gunst J, Vander Perre S, Declerck E, Pauwels L, Derese I, Van den Berghe G, Langouche L (2022). Obesity attenuates inflammation, protein catabolism, dyslipidaemia, and muscle weakness during sepsis, independent of leptin. J Cachexia Sarcopenia Muscle.

[79] Goossens C, Weckx R, Derde S, Dufour T, Vander Perre S, Pauwels L, Thiessen SE, Van Veldhoven PP, Van den Berghe G, Langouche L (2019). Adipose tissue protects against sepsis-induced muscle weakness in mice: from lipolysis to ketones. Crit Care.

[80] Dalli J, Colas RA, Quintana C, Barragan-Bradford D, Hurwitz S, Levy BD, Choi AM, Serhan CN, Baron RM (2017). Human sepsis eicosanoid and proresolving lipid mediator temporal profiles: correlations with survival and clinical outcomes. Crit Care Med.

[81] Serhan CN (2014). Pro-resolving lipid mediators are leads for resolution physiology. Nature.

[82] Zhuo Y, Zhang S, Li C, Yang L, Gao H, Wang X (2018). Resolvin D1 promotes SIRT1 expression to counteract the activation of STAT3 and NF-κB in mice with septic-associated lung injury. Inflammation.

[83] Gu J, Luo L, Wang Q, Yan S, Lin J, Li D, Cao B, Mei H, Ying B, Bin L (2018). Maresin 1 attenuates mitochondrial dysfunction through the ALX/cAMP/ROS pathway in the cecal ligation and puncture mouse model and sepsis patients. Lab Invest.

[84] Orr SK, Butler KL, Hayden D, Tompkins RG, Serhan CN, Irimia D (2015). Gene expression of proresolving lipid mediator pathways is associated with clinical outcomes in trauma patients. Crit Care Med.

[85] Hannun YA (1996). Functions of ceramide in coordinating cellular responses to stress. Science.

[86] Wu X, Hou J, Li H, Xie G, Zhang X, Zheng J, Wang J, Gao F, Yao Y, Liu H (2019). Inverse correlation between plasma sphingosine-1-phosphate and ceramide concentrations in septic patients and their utility in predicting mortality. Shock.

[87] Pradelli L, Mayer K, Klek S, Rosenthal MD, Povero M, Heller AR, Muscaritoli M (2023). Omega-3 fatty acids in parenteral nutrition - a systematic review with network meta-analysis on clinical outcomes. Clin Nutr.

[88] Manzanares W, Langlois PL, Hardy G (2016). Intravenous lipid emulsions in the critically ill: an update. Curr Opin Crit Care.

[89] Liu YM, Wang HS (2013). Medium-chain triglyceride ketogenic diet, an effective treatment for drug-resistant epilepsy and a comparison with other ketogenic diets. Biomed J.

[90] Puchalska P, Crawford PA (2017). Multi-dimensional roles of ketone bodies in fuel metabolism, signaling, and therapeutics. Cell Metab.

[91] De Bruyn A, Gunst J, Goossens C, Vander Perre S, Guerra GG, Verbruggen S, Joosten K, Langouche L, Van den Berghe G (2020). Effect of withholding early parenteral nutrition in PICU on ketogenesis as potential mediator of its outcome benefit. Crit Care.

[92] Hirabayashi T, Tanaka M, Matsumoto T, Maeshige N, Kondo H, Fujino H (2020). Preventive effects of medium-chain triglycerides supplementation on the oxidative capacity in skeletal muscle under cachectic condition. Biomed Res.

[93] Ball MJ (1993). Parenteral nutrition in the critically ill: use of a medium chain triglyceride emulsion. Intensive Care Med.

[94] Fukazawa A, Koike A, Karasawa T, Tsutsui M, Kondo S, Terada S (2020). Effects of a ketogenic diet containing medium-chain triglycerides and endurance training on metabolic enzyme adaptations in rat skeletal muscle. Nutrients.

[95] Das Pradhan A, Glynn RJ, Fruchart JC, MacFadyen JG, Zaharris ES, Everett BM, Campbell SE, Oshima R, Amarenco P, Blom DJ (2022). Triglyceride lowering with pemafibrate to reduce cardiovascular risk. N Engl J Med.

[96] Elijah IE, Børsheim E, Maybauer DM, Finnerty CC, Herndon DN, Maybauer MO (2012). Role of the PPAR-α agonist fenofibrate in severe pediatric burn. Burns.

[97] Cree MG, Newcomer BR, Herndon DN, Qian T, Sun D, Morio B, Zwetsloot JJ, Dohm GL, Fram RY, Mlcak RP (2007). PPAR-alpha agonism improves whole body and muscle mitochondrial fat oxidation, but does not alter intracellular fat concentrations in burn trauma children in a randomized controlled trial. Nutr Metab.

[98] Puskarich MA, Kline JA, Krabill V, Claremont H, Jones AE (2014). Preliminary safety and efficacy of L-carnitine infusion for the treatment of vasopressor-dependent septic shock: a randomized control trial. JPEN J Parenter Enteral Nutr.

[99] Jones AE, Puskarich MA, Shapiro NI, Guirgis FW, Runyon M, Adams JY, Sherwin R, Arnold R, Roberts BW, Kurz MC (2018). Effect of levocarnitine vs placebo as an adjunctive treatment for septic shock: the rapid administration of carnitine in sepsis (RACE) randomized clinical trial. JAMA Netw Open.

[100] Yahyapoor F, Sedaghat A, Feizi A, Bagherniya M, Pahlavani N, Khadem-Rezaiyan M, Safarian M, Islam MS, Zarifi SH, Arabi SM (2022). The effects of l-Carnitine supplementation on inflammatory markers, clinical status, and 28 days mortality in critically ill patients: a double-blind, randomized, placebo-controlled trial. Clin Nutr ESPEN.

[101] Novak F, Borovska J, Vecka M, Rychlikova J, Vavrova L, Petraskova H, Zak A, Novakova O (2017). Plasma phospholipid fatty acid profile is altered in both septic and non-septic critically ill: a correlation with inflammatory markers and albumin. Lipids.

[102] Devaud JC, Berger MM, Pannatier A, Marques-Vidal P, Tappy L, Rodondi N, Chiolero R, Voirol P (2012). Hypertriglyceridemia: a potential side effect of propofol sedation in critical illness. Intensive Care Med.

[103] Hemphill S, McMenamin L, Bellamy MC, Hopkins PM (2019). Propofol infusion syndrome: a structured literature review and analysis of published case reports. Br J Anaesth.

